# Validation of temperature methods for the estimation of pre-appearance interval in carrion insects

**DOI:** 10.1007/s12024-015-9735-z

**Published:** 2016-01-28

**Authors:** Szymon Matuszewski, Anna Mądra-Bielewicz

**Affiliations:** Laboratory of Criminalistics, Adam Mickiewicz University in Poznań, Św. Marcin 90, 61-809 Poznań, Poland

**Keywords:** Forensic entomology, Postmortem interval, Coleoptera, Diptera

## Abstract

The pre-appearance interval (PAI) is an interval preceding appearance of an insect taxon on a cadaver. It decreases with an increase in temperature in several forensically-relevant insects. Therefore, forensic entomologists developed temperature methods for the estimation of PAI. In the current study these methods were tested in the case of adult and larval *Necrodes littoralis* (Coleoptera: Silphidae), adult and larval *Creophilus maxillosus* (Coleoptera: Staphylinidae), adult *Necrobia rufipes* (Coleoptera: Cleridae), adult *Saprinus semistriatus* (Coleoptera: Histeridae) and adult *Stearibia nigriceps* (Diptera: Piophilidae). Moreover, factors affecting accuracy of estimation and techniques for the approximation and correction of predictor temperature were studied using results of a multi-year pig carcass study. It was demonstrated that temperature methods outperform conventional methods. The accuracy of estimation was strongly related to the quality of the temperature model for PAI and the quality of temperature data used for the estimation. Models for larval stage performed better than models for adult stage. Mean temperature for the average seasonal PAI was a good initial approximation of predictor temperature. Moreover, iterative estimation of PAI was found to effectively correct predictor temperature, although some pitfalls were identified in this respect. Implications for the estimation of PAI are discussed.

## Introduction

Postmortem interval (PMI) may be estimated from entomological evidence [1, 2]. Timeline of colonization and development of insects on cadavers may be predicted with reasonable accuracy enabling inferences concerning PMI [3, 4]. In most cases, forensic entomologists use laboratory developmental data to predict the age of insects sampled from a body and based on such estimates conclude the minimum PMI [5–9]. Estimation of minimum PMI may however be supplemented with an estimate of the pre-appearance interval and with this approach PMI may be concluded [10]. PMI (i.e., minimum and maximum PMI) may also be estimated from the succession of insects [11–13]. Both approaches have been substantially developed in the last few years [14–24].

The pre-appearance interval (PAI) is an interval preceding the appearance of an insect taxon on a cadaver [10]. Its length is strongly related to temperature in some carrion insects, particularly beetles [25–28]. Although in some scenarios other factors may be important, such as repellents being present on a body [29, 30] or physical barriers limiting dispersion of attractants [17, 31, 32], most frequently temperature may be considered as the single most important factor affecting PAI. Accordingly, it was postulated that we try to estimate PAI from temperature and the exponential regression model (${\text{PAI}} = c + {e^{\left( {{b_0} + {b_1}*{\text{temperature}}} \right)}}$), in which PAI is regressed against the average ground level temperature prevailing throughout the PAI [26]. In order to estimate PAI with this model, the temperature (which is called predictor temperature, as it is predictor variable within the model) needs to be approximated in some way. It was suggested that published datasets be used to calculate the average seasonal PAI, which could then be used for the calculation of predictor temperature with the case-specific temperature records [33].

Temperature methods for PAI were only validated to a limited extent in early investigations and these early works gave conflicting results as to the accuracy of estimation [10, 27, 33]. Validation has recently been the focus of some interest and several authors have pointed to the need for validation of entomological techniques in a forensic context [20, 34–37]. In the current study we tested temperature methods for PAI, identified factors that affect accuracy of estimates, and analyzed techniques for the approximation and subsequent correction of predictor temperature.

## Materials and methods

### PAI models tested

We tested models with estimated and fixed *c* parameter for adult and larval *Necrodes littoralis* L. (Coleoptera: Silphidae), adult and larval *Creophilus maxillosus* L. (Coleoptera: Staphylinidae), adult *Necrobia rufipes* De Geer (Coleoptera: Cleridae) and adult *Saprinus semistriatus* Scriba (Coleoptera: Histeridae), as published by Matuszewski & Szafałowicz [26], and the model for adult *Stearibia nigriceps* Meigen (Diptera: Piophilidae), as published by Matuszewski et al. [28].

### PAI data used for the validation

Models were validated with PAI data from our previous studies. We used results of succession experiments from 2005 [38], 2006–2007 [39], and 2012 [40] as well as some unpublished data. The dataset covered different years, seasons, and habitats. Moreover, it represented broad range of temperatures in all taxa.

### Analyses

#### Temperature and conventional methods for the estimation of PAI

We compared temperature methods against average seasonal and monthly PAI (i.e., conventional methods) as some previous results suggested that temperature methods give less accurate estimates than conventional methods [27]. Seasonal and monthly PAI were calculated using PAI data originally used while creating the models; seasonal PAI across carcasses exposed in a given season; monthly PAI across carcasses exposed in a given month. Models with estimated and fixed *c* were tested using the local weather station temperature, retrospectively corrected according to the protocol of Archer [41]. With this protocol temperature recordings from the given area are regressed against temperature recordings from the local weather station and the resultant regression model is used to correct temperature retrospectively for the period during which it was not recorded in the given area. The protocol was used to accommodate differences in temperature between the area of interest and the local weather station. Estimates of PAI, seasonal PAI, and monthly PAI were compared with the true PAI and the relative error of estimation was calculated. Temperature and conventional methods were compared according to their error rate with the Friedman rank test.

#### Accuracy of temperature methods

Accuracy of estimation was compared across taxa using PAI data from carcasses with all taxa recorded (*n* = 24). PAI was estimated using models with estimated *c* and local weather station temperature after retrospective correction. Relative error of estimation was compared across taxa with the Friedman rank test. Moreover, absolute error of estimation (models with estimated *c* and corrected weather station temperature) was analyzed separately in each taxon. In this respect mean error rate, overestimations and underestimations (frequency and maximum values) were calculated, as they are more informative than absolute values of estimates.

In order to test whether relative error of estimation is related to the temperature which was used for the estimation or carcass mass, two separate linear regression analyses were made in a pooled dataset (all taxa included).

Effect of the quality of temperature data on the accuracy of estimation was also tested. In this respect PAI was estimated using models with estimated *c* and three kinds of temperature data: on-site temperature, corrected weather station temperature, and uncorrected weather station temperature. Estimates were compared in a pooled dataset according to their error rate and the Friedman rank test was used for this purpose.

#### Techniques for the initial approximation and subsequent correction of predictor temperature

Mean temperature for the average seasonal PAI and mean temperature for the day of insect sampling were tested as initial approximations of predictor temperature. They were compared against mean temperature for the true PAI. All temperatures were weather station temperatures after retrospective correction. Models with estimated *c* were used and error of estimation using different predictor temperature was compared in a pooled dataset with the Friedman rank test.

Iterative calculation of PAI was tested as a technique for subsequent correction of predictor temperature. With this technique the first estimate of PAI is used to produce a second approximation of predictor temperature, which is used to produce a second estimate of PAI and so on. Mean daily temperature for the day of insect sampling was used as the first (i.e., initial) approximation of predictor temperature. Relative error of estimation was compared between first, second, and third estimate in a pooled dataset using the Friedman rank test. When iterations resulted in systematic overestimation of PAI (enlargement of subsequent estimates), such cases were excluded from further analyses.

All tests were used at 5 % level of significance. All calculations were made with the Statistica 10 and Statistica Medical Set (StatSoft, Inc., 2011).

## Results

### Temperature and conventional methods for the estimation of PAI

Differences between methods in the relative error of estimation were significant in the case of adult *N. littoralis*, *N. rufipes*, and *S. semistriatus* and close to significant in the case of adult and larval *C. maxillosus* and larval *N. littoralis* (Table 1). Temperature estimates were distinctly more accurate than average seasonal or monthly PAI in five taxa, whereas in larval *C. maxillosus* and adult *S. nigriceps* they were only slightly more accurate (Table 2). Models with estimated and fixed *c* performed similarly (Table 2).

**Table 1:** Results of the Friedman rank test

Analysis	Species	Stage	*N*	*χ* ^2^	*df*	*P*
*Comparison of methods*	*Necrodes littoralis*	A	64	20.1	3	<0.001
		L	42	5.7	3	0.128
	*Creophilus maxillosus*	A	63	7.7	3	0.052
		L	61	6.0	3	0.11
	*Necrobia rufipes*	A	41	14.8	3	0.002
	*Saprinus semistriatus*	A	51	8.3	3	0.04
	*Stearibia nigriceps*	A	46	2.5	3	0.47
*Comparison of taxa*	–	–	24	41.5	6	<0.001
*Comparison of temperature data*	Pooled	–	171	32.0	2	<0.001
*Initial approximations of predictor temperature*	Pooled	–	362	45.5	2	<0.001
*Corrections of predictor temperature*	Pooled	–	268	32.0	2	<0.001

*A* adult stage, *L* larval stage

**Table 2:** Mean relative error of PAI (±SE) estimated in different insect taxa using temperature and conventional methods

Species	Stage	*N*	Exponential/estimated *c*	Exponential/fixed *c*	Average seasonal PAI	Average monthly PAI
*Necrodes littoralis*	A	64	0.324 (± 0.035)	0.321 (± 0.028)	0.473 (± 0.060)	0.534 (± 0.059)
	L	42	0.164 (± 0.021)	0.185 (± 0.022)	0.284 (± 0.040)	0.252 (± 0.038)
*Creophilus maxillosus*	A	63	0.392 (± 0.050)	0.426 (± 0.049)	0.497 (± 0.065)	0.548 (± 0.070)
	L	61	0.191 (± 0.022)	0.214 (± 0.020)	0.235 (± 0.025)	0.202 (± 0.025)
*Necrobia rufipes*	A	41	0.435 (± 0.052)	0.448 (± 0.056)	0.634 (± 0.050)	0.550 (± 0.066)
*Saprinus semistriatus*	A	51	0.332 (± 0.030)	0.321 (± 0.030)	0.512 (± 0.047)	0.518 (± 0.049)
*Stearibia nigriceps*	A	46	0.573 (± 0.072)	0.568 (± 0.073)	0.610 (± 0.088)	0.723 (± 0.116)

*A* adult stage, *L* larval stage

Exponential/estimated *c*—PAI estimated using corrected weather station temperature and exponential model with estimated *c* parameter

Exponential/fixed *c*—PAI estimated using corrected weather station temperature and exponential model with fixed *c* parameter

Average seasonal PAI—average PAI calculated across carcasses exposed in a given season

Average monthly PAI—average PAI calculated across carcasses exposed in a given month

### Accuracy of temperature methods

**Fig. 1:**
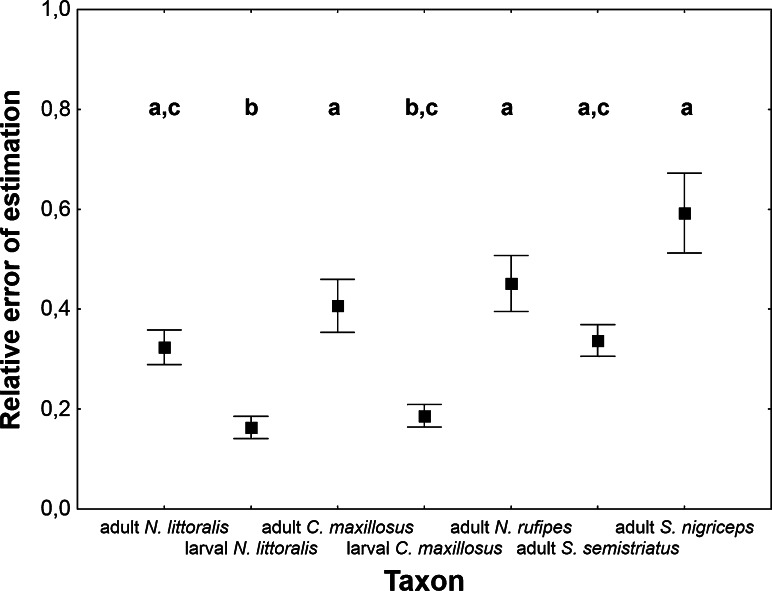
The relative error of PAI estimation in different taxa. PAI was estimated using exponential model with estimated *c* and corrected weather station temperature. *Vertical bars* represent mean ± standard error of the mean. *Different letters* denote significant differences in pairwise comparisons (*P* < 0.05)

Relative error of estimation significantly differed between taxa (Table 1). Estimates were more accurate in the case of larval than adult taxa (Tables 2, 3; Fig. 1). Average error for larval taxa was below 0.2, whereas for adult taxa it ranged between 0.3 and 0.6 (Tables 2, 3). The model for adult *S. nigriceps* performed worse than the other models (Tables 2, 3; Fig. 1).

**Table 3:** Absolute error of PAI estimated in different insect taxa using exponential model with estimated *c* and corrected weather station temperatures

Species	St.	*N*	True PAI (days)		Absolute error (days)				
			Mean	Range	Mean*	Underestimations		Overestimations	
						Frequency (%)	Maximum	Frequency (%)	Maximum
*Necrodes littoralis*	A	64	9.1	2–28	3.3	54.7	−13.7	45.3	20.1
	L	42	19.2	9–45	3.2	69.1	−16.4	30.9	4.8
*Creophilus maxillosus*	A	63	10.0	2–41	3.6	38.1	−19.2	61.9	21.7
	L	61	19.0	9–50	4.3	62.5	−23.5	37.5	21.8
*Necrobia rufipes*	A	41	18.5	5–80	8.6	31.7	−65.4	68.3	21.0
*Saprinus semistriatus*	A	51	9.1	2–29	3.2	58.8	−14.0	41.2	8.7
*Stearibia nigriceps*	A	46	8.7	2–28	3.9	34.4	−11.6	65.6	11.6

*A* adult stage; *L* larval stage

* The plus/minus sign was ignored while calculating mean

**Fig. 2:**
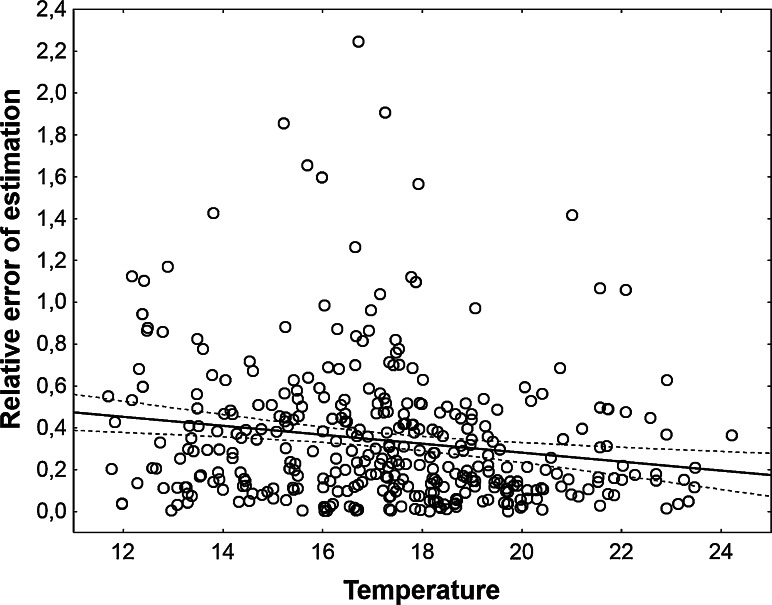
The relative error of PAI estimation plotted against temperature used for the estimation. PAI was estimated using exponential model with estimated *c* and corrected weather station temperature

**Fig. 3:**
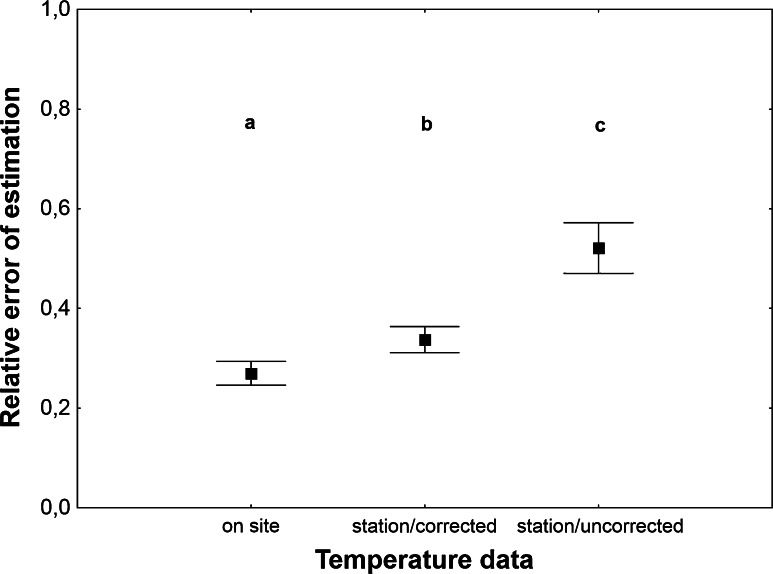
The relative error of PAI estimation using different temperature data. PAI was estimated using exponential model with estimated *c*. *Vertical bars* represent mean ± standard error of the mean. *Different letters* denote significant differences in pairwise comparisons (*P* < 0.05)

There was no significant relation of estimation error to carcass mass (linear regression, Relative error = 0.33266 + 0.000237 * Carcass mass, *t* = 0.19, *P* = 0.85, *r*^*2*^ < 0.001) and a negligible relation to temperature used for the estimation (linear regression, Relative error = 0.70945 − 0.0214 * Temperature, *t* = −3.3, *P* = 0.001, *r*^*2*^ = 0.029, Fig. 2). Error of estimation was strongly related to the quality of temperature data, as there were significant and large differences in the error rate between different kinds of temperature (Table 1). Estimates from on-site temperature were most accurate (although only little more accurate than estimates from corrected weather station temperature) and estimates from uncorrected weather station temperature were least accurate (Fig. 3).

### Techniques for the initial approximation and subsequent correction of predictor temperature

**Fig. 4:**
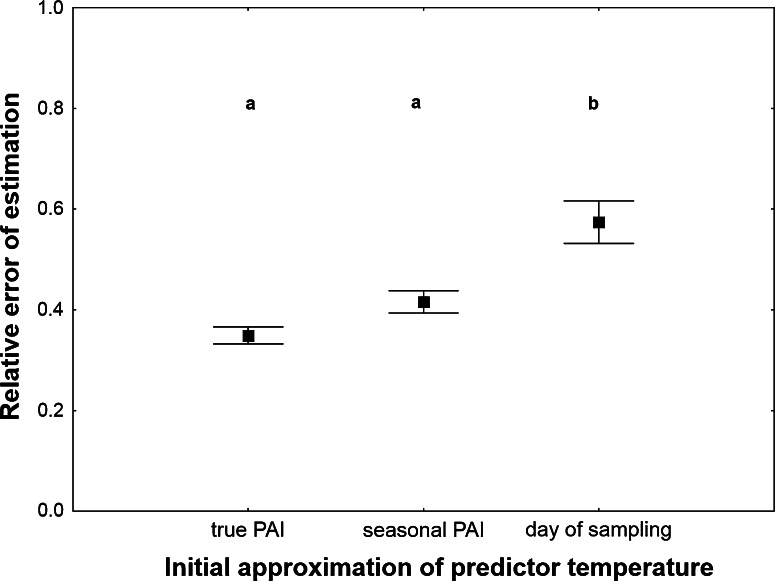
The relative error of PAI estimation using different initial approximations of predictor temperature. True PAI—mean temperature for the true PAI. Seasonal PAI—mean temperature for the average seasonal PAI. Day of sampling—mean temperature for the day of insect sampling. PAI was estimated using exponential model with estimated *c* and corrected weather station temperature. *Vertical bars* represent mean ± standard error of the mean. *Different letters* denote significant differences in pairwise comparisons (*P* < 0.05)

**Fig. 5:**
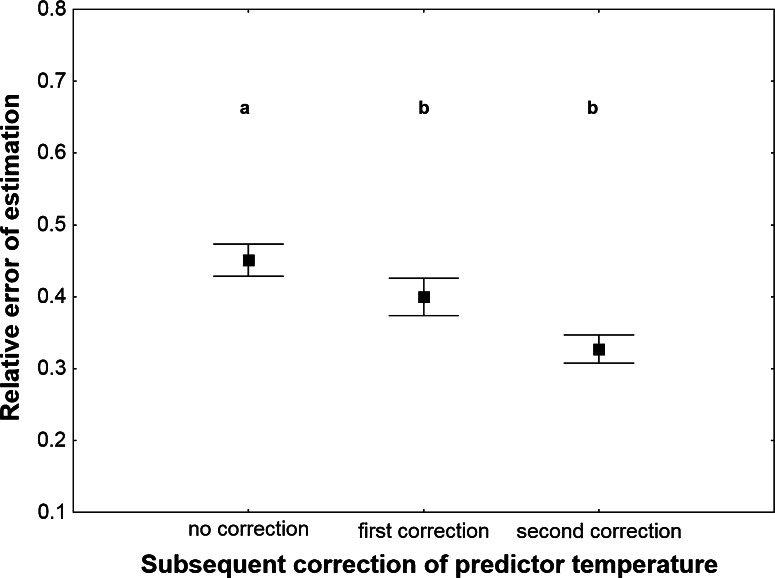
The relative error of PAI estimation with and without subsequent correction of predictor temperature. No correction—PAI estimated with the mean temperature for the day of insect sampling as a predictor temperature and no subsequent correction. First correction—PAI estimated after first correction of predictor temperature. Second correction—PAI estimated after second correction of predictor temperature. Exponential model with estimated *c* and corrected weather station temperature were used. *Vertical bars* represent mean ± standard error of the mean. *Different letters* denote significant differences in pairwise comparisons (*P* < 0.05)

Different initial approximations of predictor temperature resulted in estimates with significantly different error rates (Table 1). Mean temperature for the average seasonal PAI gave only slightly less accurate estimates as compared to mean temperature for the true PAI (Fig. 4). Mean temperature for the day of insect sampling produced estimates with a significantly higher error rate (Fig. 4), however when it was corrected iteratively, the accuracy of estimation improved (Table 1; Fig. 5). This effect was demonstrated only when cases of systematic overestimation were excluded. Systematic overestimation was particularly frequent while correcting spring temperatures. If the first PAI was overestimated and included periods of low temperature, resultant predictor temperature was underestimated and the second PAI was regularly more overestimated than the first PAI. These errors enlarged systematically when further iterations were performed. Another difficulty involved oscillating estimates. In some instances, consecutive estimates repeatedly changed from one PAI to another largely different PAI. Inspection of raw temperatures revealed that estimates start to oscillate when temperature radically changes during the relevant PAI.

## Discussion

### Temperature and conventional methods for the estimation of PAI

Temperature methods for PAI were validated to some extent by earlier studies. The simple exponential models gave estimates with a 0.24 error rate in the case of adult *N. littoralis,* a 0.19 error rate in the case of larval *N. littoralis* [33], a 0.23 error rate in the case of adult *C. maxillosus* and a 0.31 error rate in the case of larval *C. maxillosus* [10]. These methods were however not compared against conventional methods (seasonal or monthly PAI) and errors were calculated from small samples of estimates. More recently, Archer [27] tested temperature methods and summary statistics for arrival (i.e., PAI) and departure times of Australian taxa in two forensic cases, demonstrating that only summary statistics produced accurate estimates of PMI. Although it was indicated that only two cases were analyzed and both featured temperatures from the lower end of the range [27], such a weak performance of temperature methods is surprising. Contrary, current results strongly support the claim that temperature methods outperform conventional methods (average seasonal or monthly PAI) in the case of forensically-important Coleoptera. In most species of Diptera, preceding temperature is poorly related to PAI [28] and for this reason simple temperature methods seem to be insufficient for the estimation of PAI.

### Accuracy of temperature methods

Current results demonstrate that the accuracy of estimation is strongly related to the quality of the model and the quality of the temperature data used for the estimation. Models with the highest fit (for larval *N. littoralis* with *r*^*2*^ of 0.88 and for larval *C. maxillosus* with *r*^*2*^ of 0.92 [26]) revealed the lowest error rate and the model with the lowest fit (for adult *S. nigriceps* with *r*^*2*^ of 0.82 [28]) had the highest error rate. Moreover, temperature being the closest to the true temperature produced the most accurate estimates. These findings have profound implications for the practice of PAI estimation.

Firstly, only models of high quality should be used in casework. Recent studies on factors affecting the quality of PAI models revealed that field carcass studies covering a broad range of temperatures, with the frequent sampling of insects and recording of on-site temperature, warrant a high quality of PAI models [37]. Current results, however, demonstrate that performance of the models in the estimation task may substantially differ between models of comparable quality (e.g., despite a similar fit of the models, error rate for the adult *N. rufipes* was more than twice higher than error rate for the larval *N. littoralis*). These results suggest that performance with external data is related not only to the quality of a model (as measured with the fit) but also to the extent with which natural variation in PAI is represented by a model. Because estimates for larval taxa were significantly more accurate than estimates for adult taxa, it is suggested that the PAI of adult taxa is less temperature dependent than the PAI of larval taxa.

Secondly, only accurate temperature data may give accurate estimates of PAI. From this point of view protocols for retrospective correction of temperature [41, 42], models of temperature for specific environments [43] and qualitative, experience-based adjustments of temperature are of key importance. Unfortunately, there is still too little research focus in these areas. The current study demonstrates that robust protocols for the use of temperature data in forensic casework would be very beneficial for the accuracy of PAI estimation.

### Techniques for the initial approximation and subsequent correction of predictor temperature

The current study demonstrates that mean temperature for the average seasonal PAI is a good approximation of predictor temperature. Accordingly, this temperature is suggested as the best choice in casework. Moreover, iterative estimation of PAI was found to effectively correct predictor temperature and resultant estimates of PAI. However the procedure revealed its risky nature. First of all, one has to be wary of systematic overestimation (enlargement or reduction of subsequent estimates). Luckily, such faults may be easily detected, as iterations should lead to convergence of subsequent estimates around the single PAI and should not result in systematic enlargement or reduction of estimates. Accordingly, when estimates converge around a single PAI, the iterative procedure may be used safely. Moreover, in some instances the procedure may generate inconclusive results, as in the case of oscillating estimates. In such instances it is suggested iteration should not be used.

### Key points

1. The pre-appearance interval (PAI) of some carrion insects, as estimated using temperature methods, is more accurate than average seasonal or monthly PAI.
2. Accuracy of estimation is strongly related to the quality of the temperature model for PAI and the quality of temperature data used for the estimation. Models for larval stage produce more accurate estimates than models for adult stage.
3. Mean temperature for the average seasonal PAI is a good approximation of predictor temperature.
4. Iterative estimation of PAI effectively corrects predictor temperature, although caution is needed while using this procedure.
